# Opposite Effects of Two Human ATG10 Isoforms on Replication of a HCV Sub-genomic Replicon Are Mediated via Regulating Autophagy Flux in Zebrafish

**DOI:** 10.3389/fcimb.2018.00109

**Published:** 2018-04-04

**Authors:** Yu-Chen Li, Miao-Qing Zhang, Jing-Pu Zhang

**Affiliations:** Laboratory of Pharmacology, Institute of Medicinal Biotechnology, Chinese Academy of Medical Sciences and Peking Union Medical College, Beijing, China

**Keywords:** ATG10, autophagy flux, HCV subgenomic replicon, ER stress, lysosomal degradation, zebrafish model

## Abstract

Autophagy is a host mechanism for cellular homeostatic control. Intracellular stresses are symptoms of, and responses to, dysregulation of the physiological environment of the cell. Alternative gene transcription splicing is a mechanism potentially used by a host to respond to physiological or pathological challenges. Here, we aimed to confirm opposite effects of two isoforms of the human autophagy-related protein ATG10 on an HCV subgenomic replicon in zebrafish. A liver-specific HCV subreplicon model was established and exhibited several changes in gene expression typically induced by HCV infection, including overexpression of several HCV-dependent genes (*argsyn, leugpcr, rasgbd*, and *scaf-2*), as well as overexpression of several ER stress related genes (*atf4, chop, atf6*, and *bip*). Autophagy flux was blocked in the HCV model. Our results indicated that the replication of the HCV subreplicon was suppressed via a decrease in autophagosome formation caused by the autophagy inhibitor 3MA, but enhanced via dysfunction in the lysosomal degradation caused by another autophagy inhibitor CQ. Human ATG10, a canonical isoform in autophagy, facilitated the amplification of the HCV-subgenomic replicon via promoting autophagosome formation. ATG10S, a non-canonical short isoform of the ATG10 protein, promoted autophagy flux, leading to lysosomal degradation of the HCV-subgenomic replicon. Human ATG10S may therefore inhibit HCV replication, and may be an appropriate target for future antiviral drug screening.

## Introduction

Worldwide, ~150 million people are chronically infected with hepatitis C virus (HCV), a virus that leads to the development of serious liver diseases such as liver cirrhosis, liver fibrosis, and hepatocellular carcinoma (Lavanchy, 2011; Chung and Baumert, 2014). HCV, a member of the Flaviviridae family of sense single-stranded RNA viruses, has four structural proteins (core, E1, E2, and p7) and six non-structural proteins (NS2, NS3, NS, NS4B, NS, and NS5B), are encoded by the 9.6 kb HCV genome (Gale and Foy, 2005; Moradpour et al., 2007). Non-structural protein 5B (NS5B) is an HCV RNA-dependent RNA polymerase. NS5B recognizes specific RNA 3′UTR sequence of HCV RNA genome and synthesize the corresponding antisense RNA strands, which then act as templates for the amplification of multiple copies of sense RNA strands for HCV virion assembly (Gates et al., 2004; Suzuki et al., 2007). Therefore, NS5B is a popular focus of drug-target studies. Recent studies have shown that NS5B, not only acts as the HCV RNA polymerase, but also interact with host factors and resists the host immune response (Yu et al., 2012; Zhao et al., 2017). Autophagy is a host physiological mechanism that may be used by host to control cellular homeostasis. Intracellular stresses are symptoms of and responses to dysregulation of cellular physiological process involved in metabolism disorders, infection of virus or bacteria, cancers, traumas and so on, which typically include endoplasmic reticular (ER) stress and mitochondrial stress. Autophagy alleviates the stresses. Autophagy-related genes (*atg*) regulate the autophagic process; 31 *atg* genes are known to date (Klionsky et al., 2008). Among these genes, MAP1LC3B/LC3B (microtubule-associated protein 1 light chain 3) including LC3B-I and LC3B-II (formed by conjugated to phosphatidylethanolamine) are as an autophagy marker, the ratio of LC3B-II to LC3B-I, or alone LC3B-II level represents positively the level of autophagosome formation. The level of P62 (SQSTM1) degradation is used to evaluate the selective autophagy of ubiquitinated aggregates. Level of P62 and LC3II/LC3I are used to represent the overall degree of intracellular autophagy flux (Bjørkøy et al., 2005; Mizushima and Yoshimori, 2007). Recent studies have indicated a close relationship between autophagy and HCV replication (Dreux et al., 2009; Guévin et al., 2010; Zhao et al., 2017). In healthy organisms, virus infection activate the autophagic process, which typified by the formation of a double-membraned autophagosome and subsequently the virus can be eliminated by autophagolysosoms (Dreux et al., 2009; Taylor and Jackson, 2009). However, studies also have shown that HCV impairs the autophagy process and uses the double-membraned autophagosome as viral replication site for self-amplification (Taylor and Jackson, 2009; Guévin et al., 2010). Although it has been shown that HCV activates autophagy through ER stress, it remains unclear precisely how autophagy benefits HCV (Mizushima, 2007; Sir et al., 2008a,b; Ke and Chen, 2011a,b; Wang et al., 2014). In a previous study, we found that human autophagy-related gene *atg10* has two transcripts encoding two protein variants. In a HCV subreplicon cell model, we showed that human ATG10 (the long atg10 transcript) mediates Atg12-Atg5 conjugation in the early autophagosome formation and promoted replication of the HCV; while hATG10S (the short ATG10 transcript) significantly suppressed replication of the HCV subreplicon in HepG2 cells (a hepatocellular carcinoma cell line) (Zhao et al., 2017). Although these finding provided new insights into host defense mechanisms and compromise during viral invasion, further *in vivo* supporting data was required.

Up to now, HCV models have two categories, cell models and animal models. The cell models were generally founded in Huh 7 and in Huh 7.5 cell lines that are high permissiveness for HCV and 1b replication due to the cells with impaired interferon signaling (Blight et al., 2002). However, they are not applicable for research on immunology-associated antiviral issues, thus, some HCV cell models were also founded in HepG2 cell line or others (Jammart et al., 2013; Thomas and Liang, 2016). Respecting HCV animal models, chimpanzees and Tree shrews have proven to be the most useful model for the study of viral hepatitis based on their high genetic similarity with humans. However, these animals are not practical for routine use due to their limited supply and high cost (Thomas and Liang, 2016). Mice models were mostly reported to be constructed by xenotransplantation and genetic humanization. The former models are chimera and have advantages in supporting HCV replication and allowing viral kinetics and host immune studies in mice; but their disadvantages mainly are immunosuppression and HLA mismatch (Hughes and Rosen, 2009). The later models generally were transgenic models with human gene promoters directing production of HCV structure proteins or non-structure proteins in adenoviral vectors, which have intact intrinsic immunity and are available to research on interaction of viral proteins with host immunity response (Thomas and Liang, 2016). However, whether the pathogenesis of the HCV infection in mice resembles it in human still is unclear and their sustained virological response (SVR) *in vivo* were uncertain (Vercauteren et al., 2015). Thus, some alternative and small *in vivo* models are required for a more in-depth study of HCV pathogenesis and for high-throughput screening of antiviral lead compounds.

Zebrafish (*Danio rerio*) have been widely used as an animal model for the study of human diseases. We previously reported zebrafish as suitable models for HCV studies (Ding et al., 2011; Zhao et al., 2013), in which we established a simple and sensitive HCV-subreplicon model focusing on the essential elements of HCV replication, 5′UTR, CORE, 3′UTR, and NS5B. Using this model, we can easily detect actions of host factors or chemicals on only the HCV-replication associated genes and proteins. Here, based on our previous study, we created a liver-specific HCV subreplicon model in zebrafish, and investigated differences in the effects of the long (hATG10) and short (hATG10S) isoforms of human ATG10 on HCV-subreplicon replication in the zebrafish HCV model.

## Materials and methods

### Gene constructs

**Figure 1:**
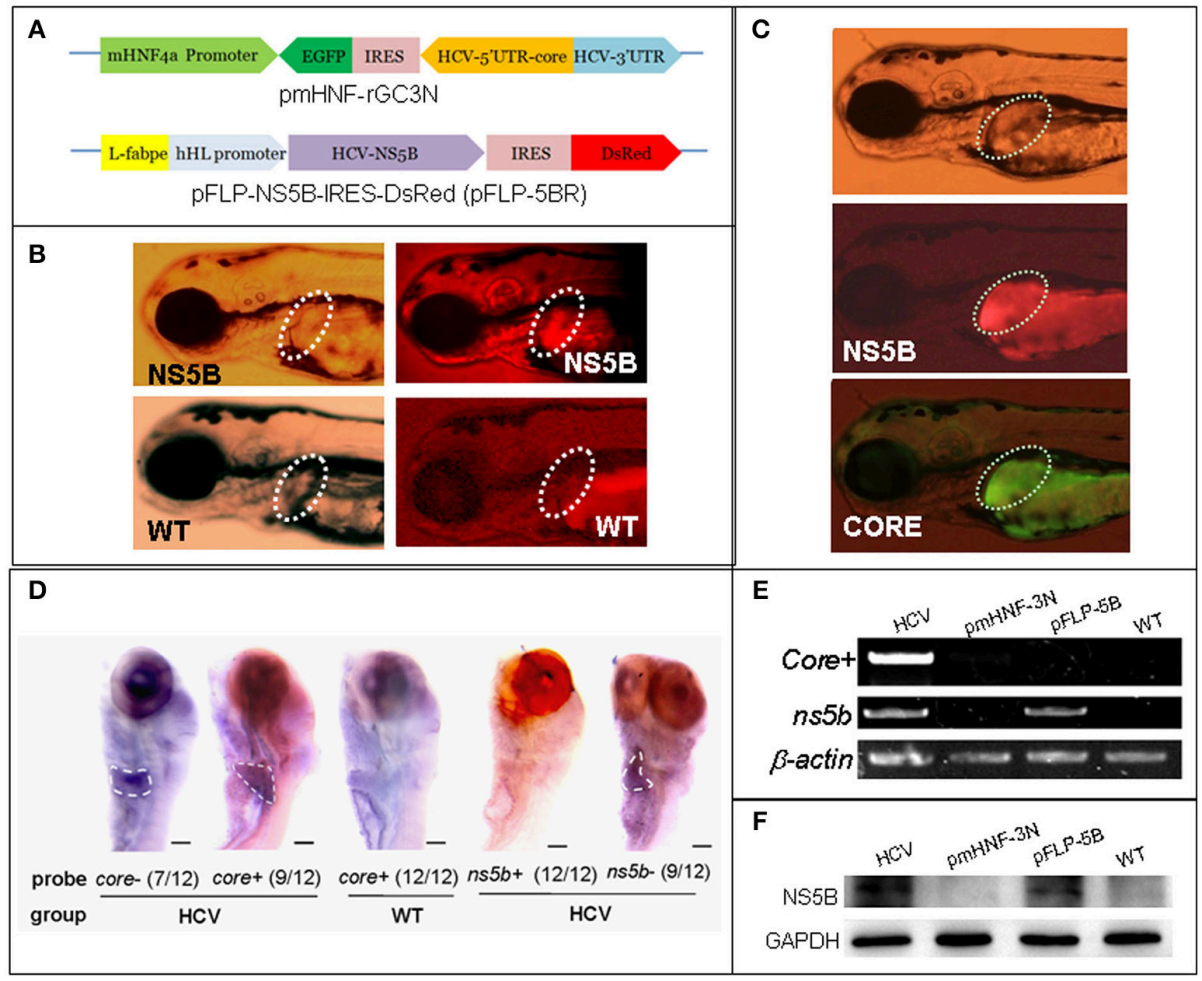
A zebrafish model of liver-specific HCV sub-replicon. **(A)** Cartoon representation of the structures of the two HCV sub-replicon vectors. The HCV sub-replicon was composed of both vectors pmHNF-3N and pFLP-5B. pmHNF-rGC3N was constructed with a mouse HNF4a promoter sequence and a complementary cDNA sequence of gfp-IRES(EMCV)-core-5′UTR fused cDNA that was reversely inserted at the downstream of the mHNF promoter and followed by a sense HCV 3′UTR sequence. Vector pFLP-5BR was consisted with zebrafish liver-*fab* enhancer and human HL promoter sequence as the transcription regular elements, followed by HCV RNA polymerase cDNA (NS5B), IRES2 (EMCV), and RFP cDNA. **(B)** NS5B expression in the livers of zebrafish larvae injected with pFLP-5BR compared to uninjected wild type fish (WT). Four-days post fertilization larvae were detected using a white light, and fluorescent RFP filter (556 nm excitation, 586 nm emission) under a fluorescence microscopy. **(C)** Fluorescence image of a live zebrafish larva at 4 days post fertilization (dpf) showing liver-targeted expression of the HCV subreplicon. Photos taken under a fluorescence microscopy using a GFP filter (480 nm excitation, 505 nm emission) and a RFP filter (556 nm excitation, 586 nm emission). Green fluorescence indicates the HCV core protein and red fluorescence indicates the HCV NS5B protein. **(D)** Whole mount *in situ* hybridization was used to detect the HCV subgenome model. The probe *core*- (HCV *core* antisense RNA) identified the sense strand of HCV *core* RNA, representing a relative replication level of the HCV subreplicon; the *core*+ probe (HCV core sense RNA) identified the antisense strand of core RNA, representing a relative transcription level of the HCV subreplicon directed by the mHNF4a promoter; the *ns5b*+ probe identified antisense strand of *ns5b* RNA (as a negative control); the *ns5b*- probe identified sense strand of *ns5b* RNA transcribed by hHL promoter. Scale bars in D represent 150 μm. **(E)** RT-PCR for the HCV *core*+ replication and *NS5B* transcription in the HCV sub-replicon. *core*+, the sense strand of HCV *core* RNA, representing products of the HCV subreplicon replication. *ns5b, ns5b* transcription level. β*-actin* was used as a loading control. WT, wild type; both pmHNF-3N and pFLP-5B larvae were used as two negative controls for the HCV model, respectively. **(F)** Western blotting confirmed that NS5B protein was produced only in the larvae injected with the HCV model plasmids (co-injection of pmHNF-3N and pFLP-5B) and with NS5B expression vector.

Liver-specific HCV subreplicon vectors were constructed based on the previously identified p5BR and prGN3N plasmids (Ding et al., 2011). To construct the two gene constructs that constitute a liver-specific HCV subreplicon, the CMV promoter in prGC3N (Ding et al., 2011) was replaced by the mouse hepatocyte nuclear factor 4a (mHNF4a) promoter, naming as pmHNF-rGC3N; and zebrafish L-fabp enhancer and human hepatic lipase promoter (FLP; Zhao et al., 2013), replaced the CMV promoter in the HCV-NS5B expression vector (p5BR; Ding et al., 2011), naming as pFLP-5BR (Figure 1A). The liver-specific HCV subreplicon zebrafish model was established via the co-injection of the two gene constructs. Human autophagy-related gene *atg10* and *atg10s* coding sequences (CDS) were cloned from HepG2 cell line and constructed into vector pIRES2-EGFP (Zhao et al., 2017). All the gene constructs were confirmed with DNA sequencing.

### Animal administration

Adult zebrafish (*Danio rerio*) AB line were obtained from Dr. Anming Meng (Tsinghua University, Beijing, China). Zebrafish Fish were maintained in a controlled environment at 28°C with a 14-h light/10-h dark cycle at 28°C (Ding et al., 2011). Our study was carried out in accordance with the recommendations of the Chinese Ministry of Science and Technology (the Regulation for the Management of Laboratory Animals). The study protocol complies with the Ethics of Animal Experiments guidelines set out by the Institute of Medicinal Biotechnology of the Chinese Academy of Medical Sciences. All the zebrafish experimental protocols were approved by the Committee on the Ethics of Animal Experiments of the Institute of Medicinal Biotechnology, Chinese Academy of Medical Sciences (IMBF20060302), which is in accordance with the NIH Guidelines for the Care and Use of Laboratory Animals (http://oacu.od.nih.gov/regs/index.htm).

### Microinjection and examination with fluorescence microscopy

Following our previous protocol (Ding et al., 2011) with minor modifications, a certain number of zebrafish embryos at 1–4 cell-stage were co-injected with a mixture of 1–5 ng/ml pmHNF-rGC3N and 1–5 ng/ml pFLP-5BR. The larvae positive for both Green Fluorescent Protein (GFP) and Red Fluorescent Protein (RFP) fluorescence were observed at 4 days post fertilization (dpf), under fluorescence microscopy. We used a 480 nm excitation wavelength and 505 nm emission wavelength to measure GFP, and a 556 nm excitation wavelength and 586 nm emission wavelength to measure RFP.

To co-injection the ATG10 and HCV subreplicon plasmids, we mixed the hATG10 and hATG10S expression plasmids with the two model plasmids pmHNF-rGC3N and pFLP-5BR at a 1:1:1 ratio. The two different mixtures were co-injected into zebrafish embryos at the 1–4 cell stage. The injected embryos developed until 8 dpf, and then the larvae were collected for detection.

### RT-PCR

At 8 dpf, both the uninjected and the pmHNF-rGC3N/pFLP-5BR co-injected zebrafish larvae were collected for the gene transcription detection with RT-PCR. Briefly, larvae total RNA was isolated with Trizol Reagent (Sigma, USA) from 50 larvae each group. We synthesized cDNA1st from 1 μg of total RNA using AMV reverse transcriptase (Promega, USA), and amplified target genes involving in ER stress and HCV infection, using RT-PCR with Taq polymerase (TaKaRa, Japan). The PCR primers were given in Table 1. The RT-PCR products were tested using electrophoresis on a 1.0% agarose gel. β*-actin* was used as a loading control.

**Table 1:** PCR primers in this study.

**Gene**	**Primer F sequence (5′-3′)**	**Primer R sequence (5′-3′)**
*core*	TCCTCTTGGCTCTGCTGTC	TCACCTTGATGCCGTTCTT
*ns5b*	GCTCGCCTTATCGTATTCC	AGTCGTCAGCACGCCAC
*rasgbd*	ATCCCTCAACTTCCCACC	TCTGCCTGCTCCACCTC
*argsyn*	GACAGGACGAGGACTTTG	TGACGGGAACAGGAATG
*Scaf2*	CTCTTGCGTCTACAGGG	GCTCAGCGGTTTCTATT
*Igr5*	GACAGGACGAGGACTTTG	GGTCTGAGTGAAGAGGGA
*bip*	AGGAGAGTGTTGGGACAGTGATT	TGATGAAGTGCTCCATGACGC
*chop*	TGGTAAACGGAGGCGCTG	CGTTTTCCGTTGAGCTCCA
*atf4*	CGCTCAGTTTGGCAAAATGG	GTGAATCCTCAGAGTCACACGAC
*Atf6*	TGTGGACAGGTTGGAGGAGG	CTCCGTCTGATTGACCCGC
*p62*	CCTGGGTTTCCGTTCACT	TACTTTGGTCCGCTTTCC
*lc3*	AAAGGAGGACATTTGAGCAG	AATGTCTCCTGGGAAGCGTA
*hatg10*	ATGGAAGAAGATGAGTT	TTAAGGGACATTTCGTTCATC
*β-actin*	AATCCCAAAGCCAACAGA	GATACCGCAAGATTCCATAC

### Drug treatment

3MA (3-Methyladenine) and CQ (Chloroquine diphosphate salt) were obtained from Sigma (Shanghai, China). We exposed 5-dpf zebrafish larvae (both un-injected and HCV subreplicon injected) to either 5 mM 3MA (*n* = 50) or 50 μM CQ (*n* = 50). Both groups of larvae were incubated for an additional 3 days (to 8 dpf) and then collected for analysis.

### Whole mount *in situ* hybridization (WISH)

The protocal was mainly followed our previous study (Ding et al., 2011) with minor modification. Briefly, c*ore* and *ns5b* antisense RNA probes were synthesized using DIG RNA Labeling Kit (Roche Diagnostics Scandinavia AB, Bromma, Sweden). We fixed 8-dpf larval zebrafish (*n* = 30) with 4% paraformaldehyde for 10 h at 4°C and washed the larvae with PBST, and decolorized with hydrogen peroxide (H_2_O_2_). Then the larvae were treated with proteinase K to increase penetrability and with DNase I to diminish DNA interference, respectively. The larvae were pre-hybridized at 65°C for 4 h, and then hybridized with the RNA probe at 65°C overnight. Free probes were removed from the larvae with 0.2 × SSC washing; the larvae were then incubated with anti-Dig-AP (Roche) at 4°C overnight. Next, we washed the larvae with PBST, and colorized them with BCIP/NBT for 30 min. Colorization was stopped with another PBST washing. The stained whole-mount larvae were observed in glycerol and visualized under an Olympus SZX16 stereomicroscope, and photographed pictures with a Canon 450D camera.

### Western blotting

Following our previous protocol (Ding et al., 2011) with modification, larval zebrafish proteins were extracted with lysis buffer and separated using 12% SDS-polyacrylamide gel electrophoresis. The protein bands were transferred onto a nitrocellulose membrane and blocked the membrane with TBS containing 5% skim milk. The membranes were incubated either with mouse anti-LC3 antibody (M186-3; MBL Biotechnology, Inc.) at 1:1,000 dilution or with rabbit anti-P62 antibody (PM 045; MBL Biotechnology, Inc.) at 1:2,000 dilution in TBS containing 1% skim milk; then the membrane was washed and incubated with secondary antibodies (HRP-conjugated goat anti-mouse or goat anti-rabbit IgGs; both 1:2,000 dilutions; Zhongshanjinqiao Co. China) for 2 h at RT. Chemiluminescent signals were detected using the Supersignal H West Pico chemiluminescent substrate (Thermo) with a GE Imaging System (GE Corporation).

### Data treatment

RT-PCR and western blotting were performed at least three times; these redults are expressed as mean ± *SD* (standard deviation) in histograms showing the relative intensity of bands as normalized to loading controls. Significance differences in our results were calculated using a one factor analysis of variance (ANOVA) in GraphPad Prism software. *P* < 0.05 were considered significant.

## Results

### Construction and identification of a liver-specific HCV sub-replicon in zebrafish

In order to construct liver-specific HCV subreplicon, we replaced CMV promoters (Ding et al., 2011) with a mouse HNF4a promoter in the HCV-subreplicon vector (named as pmHNF-rGC3N), and with a zebrafish L-fabp enhancer and a human hepatic lipase promoter in the HCV-NS5B expression vector (called as pFLP-5BR; Figure 1A). The validity of the two constructs were investigated by injection of the two plasmids into zebrafish embryos. The expression of NS5B in the liver of pFLP-5BR injected zebrafish was observed compared with wildtype (WT) larvae (Figure 1B); and then replication of the HCV subreplicon were observed simultaneously, signed with both green signal of GFP for the subreplicon amplification (pmHNF-rGC3N) and red signal of RFP for NS5B expression (pFLP-5BR), in the liver of the two plasmid co-injected zebrafish liver (Figure 1C). Then, whole mount *in situ* hybridization (WISH) was done to confirm liver-targeting of the HCV subreplicon action. Both core and NS5B positive signals primarily appeared in liver of the co-injected zebrafish larvae (Figure 1D). Both RT-PCR and western blot results indicated that NS5B expressed only in the zebrafish injected with pFLP-NS5B and pmHNF-3N plus pFLP-NS5B (the model group; Figures 1E,F). HCV *core* sense RNA (representing the HCV replication product) was detected only in the HCV model group (Figure 1E), indicating that the HCV subreplicon can self-replicate itself in dependence on HCV NS5B activity in the zebrafish. These results indicate the validity of the *in vivo* model of liver-specific HCV subreplicon.

### Expression of host genes involved in HCV infection and ER stress increased in the zebrafish HCV-subreplicon model

**Figure 2:**
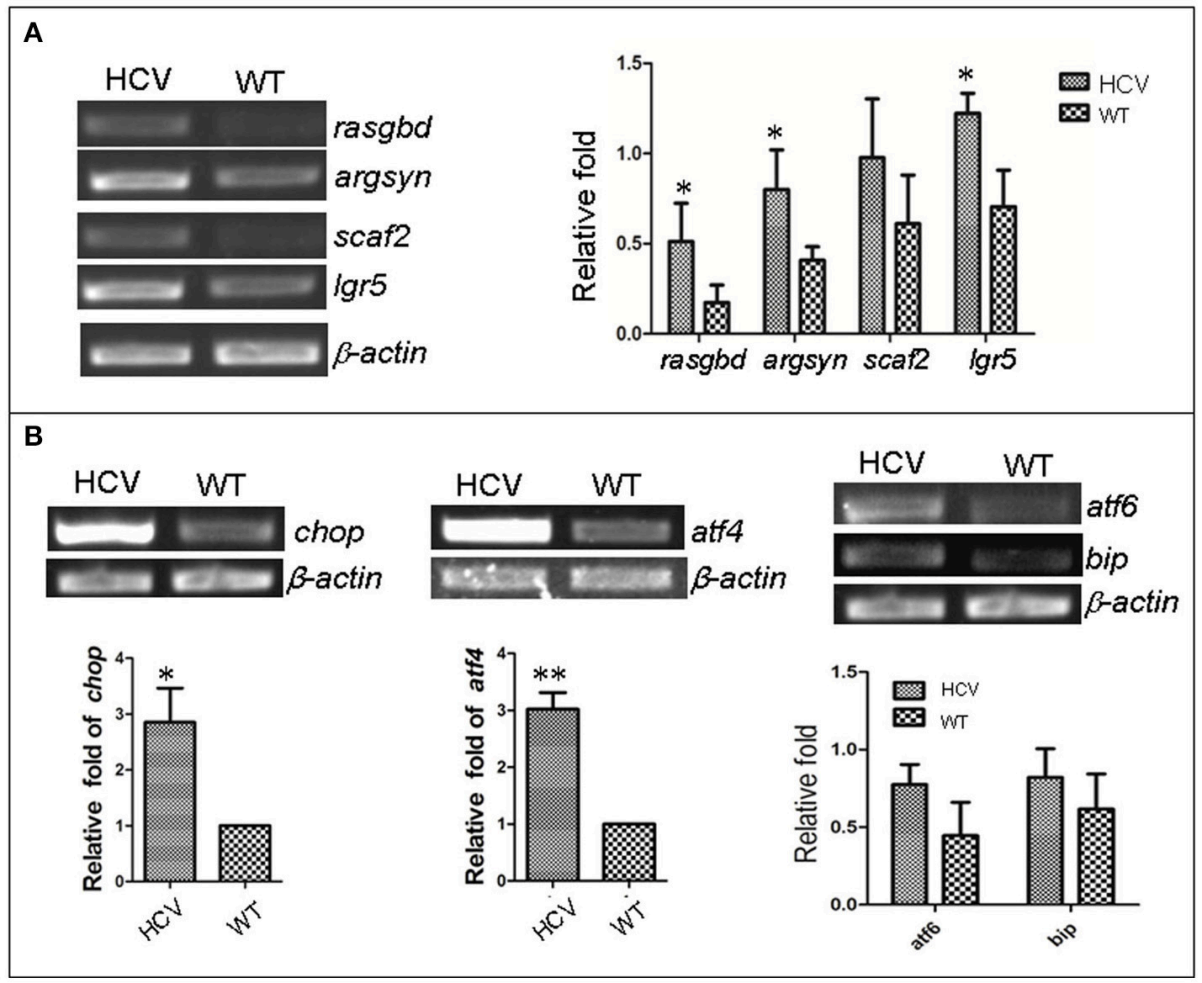
Up-regulation of HCV- and ERS-resposive host genes through the induction of the liver-specific HCV sub-replicon. Wild type (WT) and the HCV subreplicon model (HCV) zebrafish larvae were collected at 8 dpf. Transcription levels of the designated genes were tested using RT-PCR. **(A)** The transcription level of HCV-responding genes ScarF2, Leugpcr, Rasgbd and Argsyn were elevated in the model larvae as compared to the WT larvae. **(B)** UPR marker genes *chop, atf4, atf6*, and *bip* were also increased in the model larvae as compared to the WT larvae. β-actin was used as a sample loading control. The histograms are expressed as means ± *SD* from 3 independent experiments. *p < 0.05; **p < 0.001.

Previous studies have reported that HCV infection induced expression of certain host genes (Nishimura-Sakurai et al., 2010; Zhao et al., 2013) and of ER stress response in host cells (Jheng et al., 2014). To investigate these responses in the zebrafish HCV subreplicon model, we examined the expression of some HCV-responding gene by RT-PCR test, for example, Solute carrier family 2 (ScarF2), Leucine-rich repeat-containing G protein coupled receptor 5 (Leugpcr), Ras-related GTP binding D (Rasgbd) and Argininosuccinate synthetase 1 (Argsyn). Consistent with previous results (Ding et al., 2011), we found that the transcription levels of these genes in the HCV model were higher than in the WT group (Figure 2A). Unfolded protein response (UPR) is a host reaction to various unfavorable stimuli and is related to induction of ER stress. Our RT-PCR results indicated the transcription levels of UTR marker genes *chop, atf4, atf6* and *bip* in the model group were differentially elevated as compared to their levels in the WT group (Figure 2B), suggesting that the UPR was activated in the HCV subreplicon model fish.

### Influence of autophagy on the *in vivo* HCV-subreplicon

Previous studies have reported that HCV induces UPR, which in turn activates the autophagic pathway promoting HCV RNA replication in human hepatoma cells (Dash et al., 2016). We examined the role of autophagy in the zebrafish HCV-subreplicon model.

**Figure 3:**
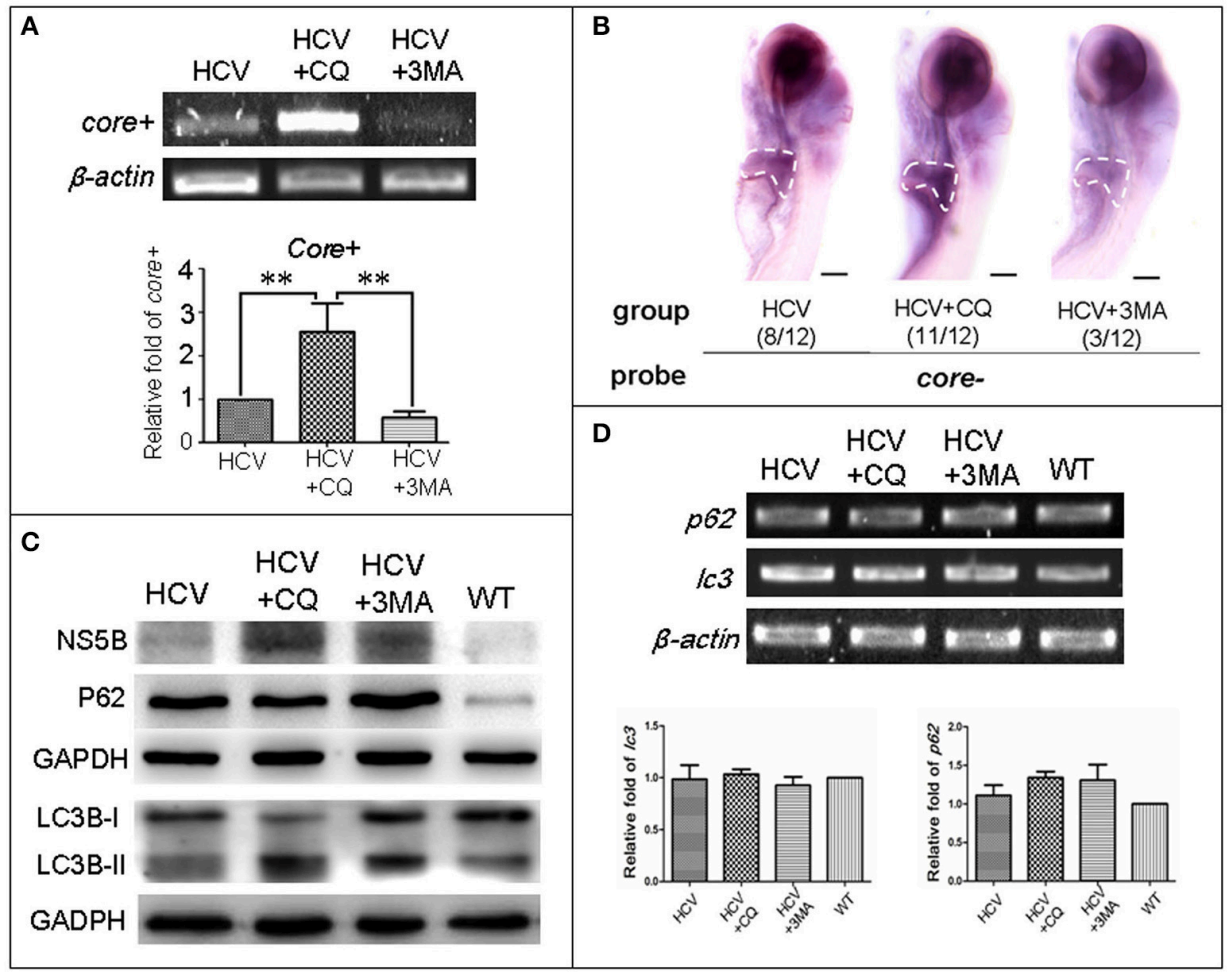
Autophagy affects the replication ability of the HCV subreplicon model. **(A)** RT-PCR test was used for quantification of the *core*+ level in unexposed HCV model larvae (HCV), HCV model larvae exposed to either CQ (50 μM) or 3-MA (5 mM). β*-actin* was used as a sample loading control. Histogram shows relative *core*+/β*-actin* ratios from three independent experiments. Data are expressed as means ± *SD*. **p < 0.01. **(B)** Whole Mount *in situ* Hybridization (WISH) indicate that the strongest core+ signal is in HCV model larvae exposed to CQ, the second one in the unexposed HCV model larvae and the lowest in the HCV model larvae exposed to 3-MA. The fraction numbers in brackets show the positive number and the total number of larvae being observed in the three groups. Scale bars represent 150 μm. **(C)** Western blot showing the protein levels of HCV-NS5B and autophagy flux marker P62 and LC3B-II/I in the unexposed HCV model larvae (HCV), the HCV model larvae exposed to either CQ (50 μM) (HCV+CQ) or 3-MA (5 mM) (HCV+3MA), and wild type larvae (WT). GADPH was used as a sample loading control. **(D)** Transcription levels of *p62* and *lc3b* genes were detected via RT-PCR tests, and were not significantly different across the four groups of larvae. All larvae were 8-dpf.

First, we used two classic inhibitors of autophagy, CQ and 3-MA, to investigate the effect of autophagy on replication of the HCV model. Using a HCV-core^−^ dependent RT-PCR detection, we found that HCV-*core*^+^ level was significantly increased in the HCV model larvae exposed to CQ and decreased in the HCV model larvae exposed to 3-MA, compared to the unexposed HCV model larvae (Figure 3A). These findings were consistent with those in WISH experiment: the highest HCV-core signal was observed in the liver of the HCV model exposed to CQ, the second HCV-core level was in the liver of the HCV model larvae, and the lowest HCV-core signal in the liver of HCV model exposed to 3-MA (Figure 3B). These findings indicate that CQ increases the replication ability of the HCV model, while 3-MA decreases it.

Next, we examined the relationship between autophagy flux and HCV replication in the HCV model. HCV NS5B protein increased in the HCV model larvae exposed to either CQ or 3MA, as compared to the unexposed HCV model larvae (Figure 3C). The results indicate that the inhibition of autophagy increases NS5B protein. A high LC3B-II/LC3B-I ratio is regarded as a positive marker for autophagosome formation. This ratio was the highest in the model larvae exposed to CQ, moderate in the model larvae exposed to 3MA and in unexposed HCV model larvae; and low in WT larvae. P62 protein levels were distinctly higher in the three groups of the unexposed HCV model, HCV model exposed to either 3-MA or CQ than in the WT group. We used RT-PCR to quantify transcription level of *p62* and *lc3b* in the unexposed HCV model, HCV model exposed to either 3-MA or CQ and WT group; no significant differences we found among the four groups (Figure 3D). This indicated that the differences in the LC3B-II/LC3B-I ratio and P62 protein level across the four groups are not related to their gene expression but may be related to impaired autophagy. Thus, an autophagy flux blockage or a restriction of the autophagolysosomal degradation function may facilitate HCV replication *in vivo*; but inhibition of autophagosome formation limits HCV replication *in vivo*, despite the presence of NS5B.

### HCV subgenome replication and autophagy flux were differentially regulated by the human ATG10 isoforms

**Figure 4:**
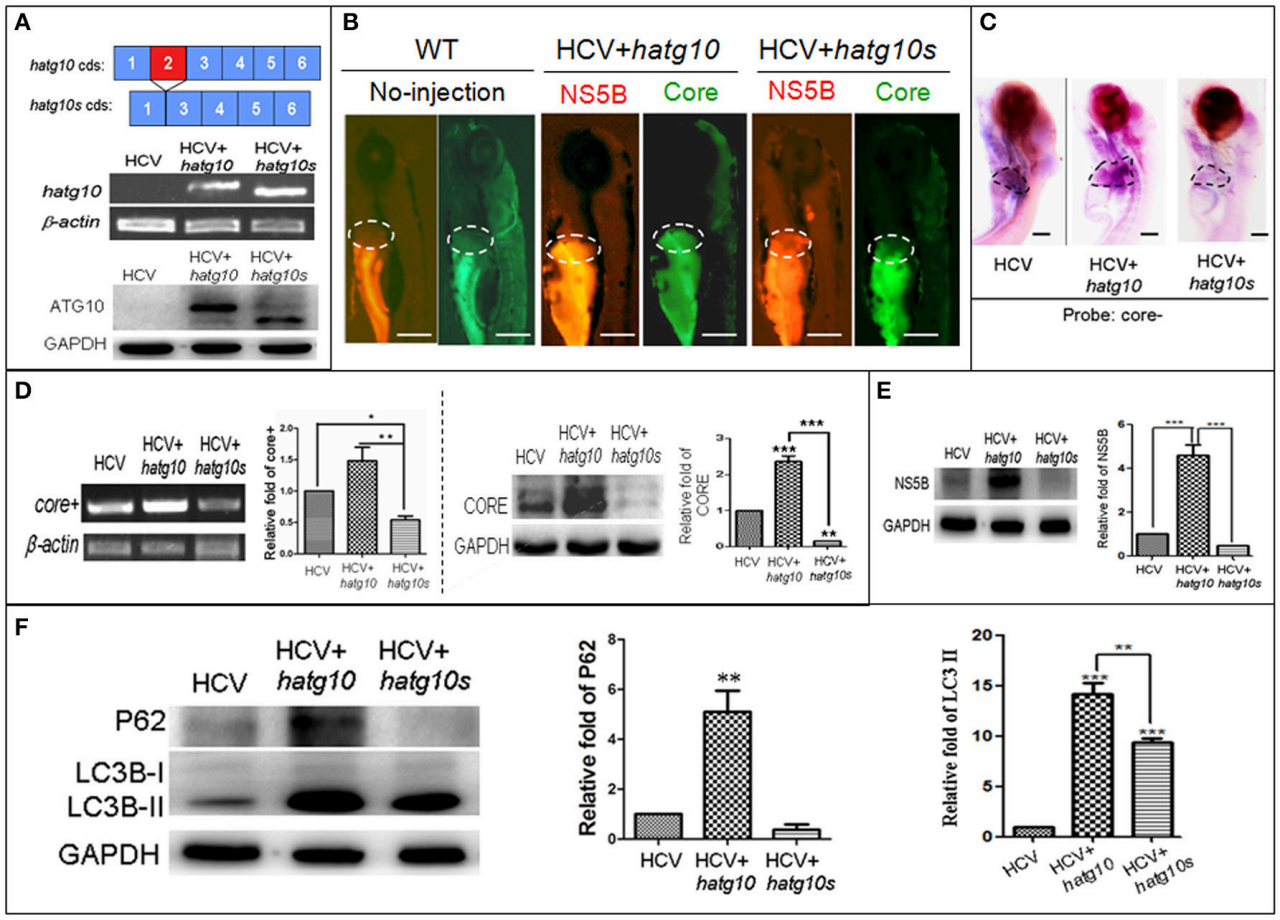
Different effects of two human autophagy related ATG10 and ATG10S on the HCV subgenomic replication and on autophagy flux in zebrafish larvae. **(A)** Diagrams show the coding regions of both transcripts of the human atg10 gene exons (upper panel). The HCV model larvae were injected either hATG10 expression vector or hATG10S expression vector separately at 1–4 cell stage and collected at 8 dpf for RT-PCR (middle panel) and western blotting (lower panel) detection. The results identified expression of hATG10 and hATG10S in the HCV subreplicon model zebrafish larvae. **(B)** Living fluorescence imaging shows variable level of the HCV subreplicon in liver of 5 dpf larvae affected by the two hATG10 isoforms. The green fluorescence represents HCV-core protein and the red fluorescence for HCV NS5B protein. Scale bars represent 280 μm. **(C)** WISH was used to detect the replication level of the HCV subreplicon by antisense HCV-core RNA probe (core-) in the three groups of no-ATG10 injected HCV model larvae (HCV), *hatg10*-injected HCV model larvae (HCV+*hatg10*) and *hatg10s*-injected HCV model larvae (HCV+*hatg10s*). The scales represent 150 μm. **(D)** RT-PCR (left panel) and western blotting (right panel) tests indicate the levels of HCV-*core*+ RNA and CORE protein changed differentially by hATG10 and hATG10S in the HCV model larvae, as compared to the no-ATG10 injected model larvae (8 dpf). β*-actin* and GAPDH were used as sample loading controls. The histograms show relative *core*+/β*-actin* ratios and CORE/GAPDH ratios, respectively, from three independent tests. Data are expressed as means ± *SD*. *p < 0.05, **p < 0.01. **(E)** Western blotting results show the level of HCV NS5B protein regulated differentially by ATG10 and ATG10S in the HCV model larvae, as compared to the no-ATG10 injected model larvae (8 dpf). GADPH was used as a loading control. The histogram shows relative NS5B/GAPDH ratios from three independent tests. Data are expressed as means ± *SD*. ***p < 0.001. **(F)** Western blotting results show the levels of LC3B-II/I and p62 proteins being regulated differentially by ATG10 and ATG10S in the HCV model larvae, as compared to the no-ATG10 injected model larvae (8 dpf). GADPH was used as a loading control. The histograms show relative LC3-II/GADPH and P62/GADPH ratios from three independent experiments. Data are expressed as means ± *SD*. **p < 0.01, ***p < 0.001.

In a preview studies we identified two transcripts of human autophagy related gene 10 (ATG10): a long transcript (hATG10, containing 220 amino acids) and a short transcript (hATG10S, containing 184 amino acids); and they presented differential effects on the HCV subreplicon. hATG10 can promote and hATG10S suppress the HCV subreplicon via regulating autophagy flux in HepG2 cell (Zhao et al., 2017). Here, we verify both hATG10 and hATG10S also have the same effect on the replication of the *in vivo* HCV subreplicon model. Our results showed that both human *atg10* long transcript and short transcript were overexpressed in the HCV model larvae, via injecting *hatg10* and *hatg10s* gene expression vectors, respectively (Figure 4A). The expression of hATG10 or hATG10S was inversely proportional to the HCV *core*+ level. Under fluorescence, living hATG10-expressed HCV model larvae fluoresced both strongly green (representing the HCV core protein) and strongly red (representing HCV NS5B protein) in the larval liver (Figure 4B). In living hATG10S-expressed HCV model larvae, the fluorescent response was weaker in the larval liver (Figure 4B), indicating that the different transcripts of ATG10 might have distinct roles on the replication of the *in vivo* HCV subreplicon, too. To verify this, we used WISH to detect *core*+ level in the larvae liver. Similarly, expression of hATG10S significantly decreased *core*+ signal and expression of hATG10 increased *core*+ signal in the liver of the HCV model larvae as compared to the HCV model larvae (Figure 4C). In addition, our RT-PCR results showed that hATG10 expression increased the levels of *core*+ RNA and NS5B protein, while hATG10S expression significantly decreased the levels of *core*+ RNA and NS5B protein in the HCV model zebrafish (Figures 4D,E). Together, these results suggest that hATG10 enhances the HCV subgenome replication, while hATG10S significantly suppresses it.

In a previous study, we also showed that human ATG10 isoforms affected autophagic flux differentially in the HCV model *in vitro* (Zhao et al., 2017). To determine whether the expression of the hATG10 isoforms also resulted in the similar change in autophagic flux in zebrafish, we measured LC3B and P62 protein levels in 8-dpf larvae. As compared to the un-injected HCV model larvae, the protein levels of LC3B-II and P62 increased significantly in the hATG10-transfected HCV model larvae, while LC3B-II level increased and P62 protein decreased in the hATG10S-tansfected HCV model larvae (Figure 4F). Those results suggest that hATG10 expression enhanced incomplete autophagy process in zebrafish larvae, while hATG10S promote a complete autophagy process. That is, consistent with the *in vitro* model of HCV subreplicon (Zhao et al., 2017), hATG10 and hATG10S have different effects on HCV subgenome replication and autophagy flux in the zebrafish model.

## Discussion

ER is the primary intracellular site for protein synthesis and post-translation processing. ER stress is defined as the process by which various adverse stimuli affect ER functions. ER stress induces UPR, several evolutionarily conserved signaling pathways that determine cell survives or dies. ER stress and UPR play an important role in the HCV lifecycle in which autophagy is involved. Several lines of evidence, both *in vitro* and *in vivo*, convincingly suggest that HCV infection or even merely overexpression of individual HCV protein are sufficient to trigger ER stress and UPR (Vasallo and Gastaminza, 2015). The coordinated expression of the UPR mediators, ATF4 and CHOP triggers the transcription of numerous UPR target genes including autophagy-regulating genes ATG5 and LC3B (Rouschop et al., 2010; B'Chir et al., 2014). CHOP is required for induction of autophagy and for HCV RNA replication, indicating that it plays an important role in UPR-induced autophagy during HCV infection (Sir et al., 2008a; Ke and Chen, 2011a). ATF6 protein is normally present in the ER, forming a stable complex with BiP protein (Shen et al., 2005). ER stress dissociates ATF6 from BiP, allowing release of ATF6 from the ER membrane, and translocating it to Golgi apparatus for sequential proteolysis (Ye et al., 2000). ATF6 pathway activation has been shown to be important for efficient HCV RNA replication (Sir et al., 2008a; Ke and Chen, 2011a; Vasallo and Gastaminza, 2015). BiP plays a pivotal role as the master negative regulator of UPR; Bip and ATF6 were both overexpressed in patient liver biopsy and in mice HCV-infected liver (Chan, 2014). In this study, transcription of chop and aft4 increased substantially, and of aft6 and bip increased moderately, indicating that in the zebrafish HCV model, the mechanisms of UPR and ER stress activation were consistent with other HCV models.

Several genes have previously been shown to be upregulated in response to HCV infection, including *argsyn, ScarF2, lgr5*, and *rasgbd* in HCV infection model (Nishimura-Sakurai et al., 2010). Recent studies have shown that the ARGSYN protein is required for cell migration in gastric cancer cell lines (Shan et al., 2015a), and that *argsyn* expression in gastric cancer was associated with a poor prognosis (Shan et al., 2015b). In addition, inhibition of *argsyn* gene expression sensitizes lymphomas to autophagy (Delage et al., 2012). ScarF2 is a member of the well-studied facilitative glucose transporter (GLUT) family (He et al., 2009); moreover, scarF2 also is one of responders to HCV infection (Nishimura-Sakurai et al., 2010). LGR5 protein, an intestinal stem cell marker, is overexpressed in various tumors; *lgr5* gene expression was correlated with invasion and metastasis (Xi et al., 2014). LGR5 protein was also shown to regulate epithelial cell phenotype and the survival of hepatocellular carcinoma cells (Fukuma et al., 2013). These results suggest that LGR5 overexpression is associated with hepatocellular carcinoma. Rasgbd is sensitive to amino acid stimulation and influences lysosomal function by affecting the translocation of mTORC1 to lysosomal surface (Sancak et al., 2010). Thus, it is clear that the genes argsyn, ScarF2, LGR5, and rasgbd are probably associated with liver pathological process, including glycolipid metabolism disorder, hepatocellular carcinoma, and autophagy. Here, our results show that the genes argsyn, ScarF2, LGR5, and rasgbd were also overexpressed in the HCV model larvae, indicating that our HCV subreplicon model at least partly presents HCV-related pathological features in gene expression in zebrafish liver.

Typically, the HCV replicase complex are formed by NS3, NS4A, NS4B, NS5A, and HCV-RNA polymerase NS5B. NS3, NS4A, NS4B, and NS5A can assist NS5B replicase to assemble replicase machineries for HCV RNA replication via cleaving the non-structure polyprotein and via attaching on ER membrane web, etc. (Gu and Rice, 2013). However, the key players responsible for HCV RNA replication are HCV-5′UTR, 3′UTR RNA sequences and NS5B protein (Luo et al., 2003; Mahias et al., 2010; Fricke et al., 2015). So, we constructed the HCV subgenomic model. Our studies show that NS5B alone really has replication activity *in vitro* (Zhao et al., 2017) and *in vivo* (Figures 1E,F and in Ding et al., 2011); the simple HCV models are available for research the clearance of HCV products by autophagy. In this study, whether/how elimination of the HCV products, HCV RNA and NS5B protein, via autophagy *in vivo* is the focus instead of HCV NS5B expression regulation. So, the NS5B gene expression in the model was directed by zebrafish Liver-fabp enhancer and human hepatic lipase promoter for NS5B liver-specific expression in equality in the different experimental conditions. And the HCV RNA replication was presented as level of the HCV-core RNA that was amplified by HCV-5'UTR and 3'UTR dependent NS5B activity, and tested by RT-PCR using HCV-core sense-sequence dependent primer for the cDNA1st reverse transcription. Thus, existence of HCV-core RNA reflects NS5B activity and the levels of HCV-core RNA and NS5B protein represent effects of autophagy cleaning the HCV products in the HCV larvae. The testing principle for the HCV subreplicon replication was introduced in detail in our previous paper (Ding et al., 2011; Zhao et al., 2017).

This study, for the first time, reports that the opposite roles of human ATG10 and ATG10S on HCV replication and elimination of HCV products by keeping autophagic-flux open were confirmed in a vertebrate model. Though compared with our previous study (Zhao et al., 2017) this study originality is weaker and the main change is the experimental model that goes from HepG2 hepatoma cell lines to zebrafish, this is an important progress. As we know, there are considerable differences between *in vitro* models and *in vivo* models in immunity response, endocrine control and neuroregulation; these host modulation systems are important for host defending exotic pathogens. Animal models of diseases, especially, vertebrate models of diseases, can closely mimic the human defending responses to exotic pathogens; while cell models cannot due to without the integrative modulation systems. So, development of small and practical vertebrate disease models is important for a more in-depth study of HCV pathogenesis and for high-performance and preliminary screening of antiviral lead compounds.

Since 2011, HCV direct-acting antivirals (DAA) have been well-developed and presented dramatic effects on treatment of HCV infection with increase of SVR rate up to over 90% and have a shortened treatment duration to 12–24 weeks (Ahn and Flamm, 2014; Kumthip and Maneekarn, 2015). These DAA drugs are HCV protein inhibitors, such as NS5B inhibitor Sofosbuvir, HCV NS5A inhibitor Daclatasvir and NS3/4A inhibitor Simeprevir, and so on. Despite the exciting improvement achieved, drug resistance has emerged as a potential challenge (Esposito et al., 2016), which occurred on both treatment-naive patients as natural polymorphisms and who carrying DAA selected resistance-associated variants (RAVs) upon treatment failure in different viral strains and genotypes in clinic treatment. Patients who carry RAVs may not only suffer the treatment failure but also mediate transmission of the variants (Colpitts and Baumert, 2016). The variation of HCV amino acids targeted by DAA drugs caused the targets disappeared and efficacy of DAA drugs lost, leading to failure of clinical treatment (Ahmed and Felmlee, 2015; Kim et al., 2016). Thus, for resolving the drug-resistance problem, alternative strategies or ideas have emerged for new antiviral drug development; that is, besides finding continually new DAA drugs, to enhance the research and development of new host factors focusing on removing HCV products such as HCV RNA and HCV proteins no matter how changed the viral proteins are and whether DAA resistance produced. Therefore, chemicals which can activate this kind of host antiviral factors will be highly promising and complementary to DAA drugs.

It is well-known that alternative splicing of gene transcription crucially affects disease occurrence and patient outcome. Different protein isoforms resulted from one gene alternative-splicing mRNA variants may be involved in individual physiology, pathology, and tissue-specific actions (Kim et al., 2008; Ward and Cooper, 2010; Gutierrez-Arcelus et al., 2015). Numerous studies have shown that some viruses exploit host resources, circumvent host immunity, and hijack autophagy networks for viral replication and survival (Sir et al., 2008a,b; Taguwa et al., 2011; Yordy and Iwasaki, 2011; Chiramel et al., 2013; Ploen and Hildt, 2015; Dash et al., 2016). Previously, we identified two atg10 variants generated by mRNA alternative splicing: atg10 and atg10s (Zhao et al., 2017). The protein ATG10S is consisted of 184 amino acids and ATG10 is 220 amino acids long (GenBank XM_005248612.1). The two atg10 isoforms differently affected the HCV subreplicon replication, innate immunity response and the autophagy flux of the cell model of HCV subeplicon (Zhao et al., 2017). Here, we again demonstrated the different effects of human atg10 isoforms on the zebrafish HCV subreplicon model, which probably mediated by their differential action on autophagy flux. We also found that CQ, an inhibitor of the late stage of autophagy, significantly increased the HCV subreplicon replication by blocking autophagy flux; while 3MA, another inhibitor at the middle stage of autophagy, apparently decreased the HCV subreplicon replication by suppressing autophagosome formation. As the effects of hATG10 on the HCV model were similar to those by CQ, we postulate that hATG10 overexpression “obstruct” autophagy flux through its over-promotion of autophagosome maturation via mediating ATG5-ATG12 formation (Walsh and Edinger, 2010), resulting in enhancement of HCV replication on the autophagosome membrane web. In contrast, hATG10S suppresses replication of the HCV model in zebrafish, probably similar to it in the HCV cell model: hATG10S promoting autophagy flux by enhancing autolysosome formation and lysosomal degradation of the HCV subreplicon (Zhao et al., 2017).

Thus, ATG10S may be as an antiviral target for screening chemicals that can activate the host antiviral factors; these chemicals may become important complement to DAA treatment to overcome the HCV DAA-resistance.

## Author contributions

J-PZ conceived and designed the project; Y-CL and M-QZ performed the experiments and treated data; Y-CL and J-PZ wrote the manuscript.

### Conflict of interest statement

The authors declare that the research was conducted in the absence of any commercial or financial relationships that could be construed as a potential conflict of interest.
